# Structure-Composition-Property Relationships in Polymeric Amorphous Calcium Phosphate-Based Dental Composites †

**DOI:** 10.3390/ma2041929

**Published:** 2009-11-24

**Authors:** Justin N.R. O’Donnell, Gary E. Schumacher, Joseph M. Antonucci, Drago Skrtic

**Affiliations:** 1 Paffenbarger Research Center, American Dental Association Foundation, Gaithersburg, 20899, MD, USA; E-Mails: justin.odonell@nist.gov (J.N.R.O’D.); gary.schumacher@nist.gov (G.E.S.); 2 Polymers Division, National Institute of Standards and Technology, Gaithersburg, 20899, MD, USA; E-Mail: joe.antonucci@nist.gov (J.M.A.)

**Keywords:** amorphous calcium phosphate, bioactivity, cellular response, dental composite, mechanical strength, polymerization, re-mineralization, resin

## Abstract

Our studies of amorphous calcium phosphate (ACP)-based materials over the last decade have yielded bioactive polymeric composites capable of protecting teeth from demineralization or even regenerating lost tooth mineral. The anti-cariogenic/re-mineralizing potential of these ACP composites originates from their propensity, when exposed to the oral environment, to release in a sustained manner sufficient levels of mineral-forming calcium and phosphate ions to promote formation of stable apatitic tooth mineral. However, the less than optimal ACP filler/resin matrix cohesion, excessive polymerization shrinkage and water sorption of these experimental materials can adversely affect their physicochemical and mechanical properties, and, ultimately, limit their lifespan. This study demonstrates the effects of chemical structure and composition of the methacrylate monomers used to form the matrix phase of composites on degree of vinyl conversion (DVC) and water sorption of both copolymers and composites and the release of mineral ions from the composites. Modification of ACP surface via introducing cations and/or polymers *ab initio* during filler synthesis failed to yield mechanically improved composites. However, moderate improvement in composite’s mechanical stability without compromising its remineralization potential was achieved by silanization and/or milling of ACP filler. Using ethoxylated bisphenol A dimethacrylate or urethane dimethacrylate as base monomers and adding moderate amounts of hydrophilic 2-hydroxyethyl methacrylate or its isomer ethyl-α-hydroxymethacrylate appears to be a promising route to maximize the remineralizing ability of the filler while maintaining high DVC. Exploration of the structure/composition/property relationships of ACP fillers and polymer matrices is complex but essential for achieving a better understanding of the fundamental mechanisms that govern dissolution/re-precipitation of bioactive ACP fillers, and, ultimately, the suitability of the composites for clinical evaluation.

## 1. Introduction 

Despite significant reduction in the occurrence of dental caries in some segments of population, it remains one of the most prevalent diseases affecting humans. There is a continuing need to develop new approaches to prevent and repair dental tissues damaged by caries and increase the efficacy of widely used fluoride without increasing its doses and/or administration frequency because of the associated concerns regarding fluorosis [1]. Tooth demineralization/remineralization is a dynamic process that includes dissolution of calcium and phosphate ions from the tooth mineral into the saliva (demineralization) and their precipitation back into tooth structures (remineralization). If these processes are balanced, then no net mineral loss occurs. However, local decrease in pH caused by bacterial plaque, or an overall oral acidification due to the frequent intake of acidic foods and beverages disrupts the delicate demineralization/remineralization balance and often results in generalized demineralization [2]. If uncontrolled, over time this demineralization leads to tooth erosion and, in advanced cases, caries. Therefore, inhibition of uncontrolled demineralization is the primary target in anti-caries therapies. For the treatment to be clinically successful it is essential that a protracted supply of ions (calcium, phosphate and/or fluoride) needed to regenerate mineral-deficient structures be provided and that replacement of lost mineral occurs faster than in natural salivary mineralization. The use of remineralizing solutions containing calcium and phosphate ions have not been successful clinically [3], particularly in the presence of fluoride ions. On the other hand, insoluble calcium phosphates are not easily applied and do not localize effectively at the tooth surface. One of the recently proposed technologies introduces casein phosphopeptide-amorphous calcium phosphate (ACP) nanoclusters in metastable solutions. Such nanoclusters are capable of localizing at the tooth surface and have been shown to prevent caries in laboratory, animal and human *in situ* caries models [3,4,5,6]. An alternative anti-demineralization/remineralization therapy is the proposed use of polymeric composites based on ACP developed in our group. Physicochemical data [7,8,9,10,11,12] and the results of *in vitro* testing [13,14] show that ACP composites in various biostable matrices release calcium and phosphate ions in a manner that effectively buffers free calcium and phosphate ion activities and, in turn, maintains the desired state of supersaturation with respect to tooth mineral. As a result, these composites have the capacity to not only prevent the formation of new carious lesions, but also actively repair existing incipient lesions. Their main advantage is concentrating calcium and phosphate release to the site of caries attack. A wide variety of dental products could be made with only small to moderate variations in chemistry and formulation. Examples include orthodontic bonding cements, crown and bridge cements, root caries filling materials, and provisional restorations for caries control. In addition to dental applications, bioactive ACP composites formulated with biodegradable polymeric matrices may find utility as general therapeutic materials for bone regeneration/reconstruction. 

In this paper we explore how modifying the ACP filler and fine-tuning the composition of the resin matrix affects physicochemical and mechanical properties of the experimental composites and whether such an approach can be used to tailor remineralizing ACP composites for various dental applications. Critical properties of interest are degree of vinyl conversion (DVC), polymerization shrinkage (PS) and stress (PSS), water sorption (WS), ion release kinetics, biaxial flexure strength (BFS)), their adhesion to tooth substrates (shear bond strength (SBS)) and the *in vitro* cellular responses to these materials. Additionally, the comprehensive structure-composition-property relationship studies are expected to improve our understanding of the multifaceted interactions occurring at the filler/polymer interfaces thus expanding the knowledge base needed for the successful design of improved calcium phosphate-based dental composites. 

## 2. Results and Discussion 

Routinely synthesized pyrophosphate-stabilized ACP [15], the bioactive filler used to formulate polymeric ACP composites, is typically highly agglomerated with a heterogeneous particle size distribution ranging from submicrometer up to 100 to 200 µm. This intrinsic, uncontrolled agglomeration of ACP particles and the lack of strong bonding mechanisms between ACP and resin phase can hinder interfacial interaction and typically results in mechanically inferior ACP composites compared to glass-reinforced materials [9]. Moreover, the state of aggregation of ACP filler in composites is expected to have a significant effect on the ion release properties of these materials. It has been documented in the literature that metal ions in dental materials may: affect calcium phosphate precipitation and/or transformation [16,17], improve adhesive bonding of composites via chelating and/or multiple interacting with surface-active resin comonomers [18], promote bone regeneration [19, 20], regulate dental calculus formation [21], affect the free-radical polymerization of the resins [22], aid in detecting defects in the subsurface wear layer [23] and control the topical calcium fluoride deposition in bio-glasses [24]. There is also evidence that polymers containing multiple charged functional groups influence the surface properties and transformation kinetics of calcium phosphates [25,26]. Modification of ACP with either cations or polymer (polyelectrolyte) molecules is perceived as a way to alter ACP’s surface and potentially lead to a less agglomerated ACP that would also dissolve in aqueous milieu at a reduced rate. To test the above hypothesis, cations and polymers were introduced *ab initio* during the ACP synthesis and their effects on structure, composition, morphology, particle size distribution (PSD) and dissolution kinetics of the precipitated solids were assessed. Results are presented in Table 1. 

**Table 1:** Effect of cations and polymers on the particle size (median diameter; d_m_), calculated specific surface area (SSA) and water content of the precipitated ACP solids, and degree of vinyl conversion (DVC, 24 h post-cure) and the mechanical strength (biaxial flexure strength (BFS) of specimens immersed in saline for one month) of their composites formulated with ^a^EBPADMA/TEGDMA, ^b^Bis-GMA/TEGDMA or ^c^Bis-GMA/TEGDMA/ HEMA/ZrDMA resins (for the chemical names of the monomers and their corresponding acronyms please see Table 3). Indicated are mean values with one standard deviation (SD) given in parenthesis. *ACP/HAP denotes appearance of apatite crystalline peaks in X-ray diffraction spectra. nd – not determined. PAA - poly(acrylic acid); PEO – poly(ethylene oxide).

Additive	d_m_ (μm)	Structure*	SSA (m^2^/g)	Water content (%)	DVC (%)	BFS (MPa)
ilver^a^	3.5 (1.9)	ACP	1.2 (0.9)	14.0 (2.2)	63.3 (1.9)	disintegrated
Iron (II)^a^	3.8 (1.8)	ACP/HAP	1.0 (0.5)	15.4 (1.2)	65.7 (1.8)	disintegrated
Zinc^a^	1.4 (0.5)	ACP	2.7 (1.1)	16.6 (2.5)	63.7 (2.6)	48.4 (5.3)
Aluminum^a^	2.2 (1.3)	ACP	1.6 (0.8)	14.1 (2.3)	56.0 (3.3)	19.8 (4.7)
Iron (III)^a^	2.1 (0.6)	ACP/HAP	1.6 (0.8)	16.8 (2.8)	56.7 (2.6)	disintegrated
Silica ^a,b,c^	5.8 (1.6)	ACP		14.1 (1.2)	72.5 (2.5)	40.0 (9.0)
Zirconia^a,b,c^	6.7 (1.9)	ACP	0.5 (0.3)	16.1 (2.0)	80.1 (3.3)	53.4 (12.0)
PAA^b^	9.2 (1.9)	ACP	0.7 (0.1)	15.8 (1.0)	nd	34.1 (9.9)
PEO^b^	14.1 (4.7)	ACP	0.5 (0.2)	14.7 (1.2)	nd	23.4 (4.3)

The PSD data revealed the following order of decreasing d_m_ in cation-ACP series: (Si-ACP, Zr-ACP) > (Ag-, Fe(II)-, Al-, Fe(III)-ACP) > Zn-ACP. In addition to the unwanted color change due to the co-precipitation of Fe-phosphates, Fe(II)- and Fe(III)-ACP also showed the signs of an early conversion to apatite [Fourier-transform infrared (FTIR) spectroscopy and X-ray diffraction (XRD) data]. Color instability was also observed with Ag-ACP. The mechanical strength of cation composites decreased in the following order: (Zr-, Zn-ACP) > Si-ACP > Al-ACP. Ag-, Fe(II)- and Fe(III)-ACP based composites disintegrated upon immersion. In addition to the highest BFS values, Zr-ACP also showed the highest DVC of all cation-modified ACPs. Rather than reducing the particle size of the precipitating solid, the d_m_ of PAA- and PEO-ACP increased compared to cation-ACPs and the corresponding polymer-ACP composites were generally mechanically inferior to Zn-, Si- or Zr-ACP composites. Interestingly, particle morphology [scanning electron microscopy (SEM) data not shown here] and the water content [thermogravimetric analysis (TGA) results] of all modified ACPs [on average (15.4 ± 1.7) mass%] appeared unaffected by the type of additive used during the synthesis. It remains an open question whether the somewhat reduced level of the structural water in cation and polymer-modified ACPs (water content of ACP reported in literature varies typically from 17.4 to 26.1 mass% [27,28,29]) could have an effect on the WS and/or the PS of their respective composites. Although the dissolution/transformation kinetics of the cation-ACPs revealed somewhat slower transformation of ACP in Zn- and Al-modifications [30] compared to Zr-ACP, the latter was still chosen as standard filler and was used in all subsequent studies described in paper due to the higher DVC and BFS values attained in Zr-ACP formulations. Since Zn was indicated as an essential trace element with stimulatory effects on bone formation [19,20], a favorable PSD and moderately improved solution stability of Zn-ACP filler may yet be of particular interest in design of the remineralizing ACP composites intended for periodontal tissue repair or even general bone regeneration. As designed, ACP surface modification experiments provided no evidence to support the working hypothesis that cation/ACP interactions are controlled by the ionic potential (defined by the water coordination number, multiple charge and ionic radius) and polymer/ACP interactions are controlled by the structure and size of the polymer’s ionizable groups [24]. The above results suggest that other types of polymeric additives and/or surface active agents should be explored or alterations in the surface modification protocol are needed in order to fabricate ACP fillers with significantly narrower PSD and improved dispersion of such finer fillers within the polymer matrix, in order to obtain composites with enhanced mechanical performance. In an attempt to do so, we tried to modify ACP by introducing nonionic and anionic surfactants during its synthesis but with no success [31]. Model surfactants exhibited no significant effect on PSD and morphology while significantly reducing the mechanical strength of surfactant-ACP based composites compared to Zr-ACP composite controls. 

A major factor affecting the thermal, physical, and mechanical properties of polymer composites is the stability of the interface between the filler and the resin [32,33]. WS, important with respect to service performance in aqueous environments (reduction of mechanical properties, ion mobility, release of organic components that may be potent sensitive/irritative agents), is affected by the critical interfacial region between the filler particles and the matrix. An important question in this regard, is whether voids or non-bonding spaces in the filler/matrix interfaces can cause an increase in WS, ultimately leading to mechanically compromised composites. One of the generally accepted ways to improve the filler/resin interface is the use of coupling agents. A variety of different coupling agents have been developed to enhance filler/matrix interactions [34,35,36] by concomitant covalent bonding of organic functionalities with the polymer matrix and hydrolyzable silane groups with the inorganic filler. Additionally, silanol groups on adjacent silanes may form a polymer film on the surface by a condensation reaction. The silane coupling agents used in dental composites are mostly alkyloxy silanes. Many laboratories and manufacturers use proprietary methods and there is no general consensus regarding the best method of silanation. We hypothesize that an effective silane coupling agent will reduce the interfacial penetration of water between the ACP particles and polymeric matrix and, in turn, ameliorate the overall plasticization of composites. Due to differences in the molecular structure/reactivity of organic functional groups, methoxysilane agents possessing vinyl, primary amine and methacryloxy functionalities are expected to have different affinities for multilayered chemisorption on ACP surfaces. These differences in affinity will affect the stability of the silane coatings and their ability to form strong polysiloxane structures. It is assumed that the silane-coated ACP(s) will remain “transparent” for ionic transport in aqueous environments due to the low thickness and open flexible structure of the silane layers. If successfully applied, such coatings will provide sufficient chemical ACP/matrix bonding without weakening the properties of the interface in a composite formulation. The silane coupling agents are also expected to facilitate resin penetration into the surface cavities formed during filler particle aggregation. This penetration should enhance mechanical interlocking between the filler and the matrix of the resultant composite. In addition to mechanical improvement, it is also expected that the hydrolytic stability of the composites will be enhanced because of the hydrophobic nature of the silane coupling agents. To test whether silanization of ACP filler can reduce the interfacial penetration of water between the ACP particles and polymeric matrix, 3-aminopropyltrimethoxysilane (APTMS) and methacryloxypropyltrimethoxysilane (MPTMS) were applied at 2 mass% relative to Zr-ACP. Silanized filler was used to formulate composites with EBPADMA/TEGDMA/HEMA/MEP (ETHM) resin (40 mass% filler + 60 mass% ETHM resin) and composite specimens were evaluated for BFS after 1 mo immersion in saline solutions. 

**Figure 1:**
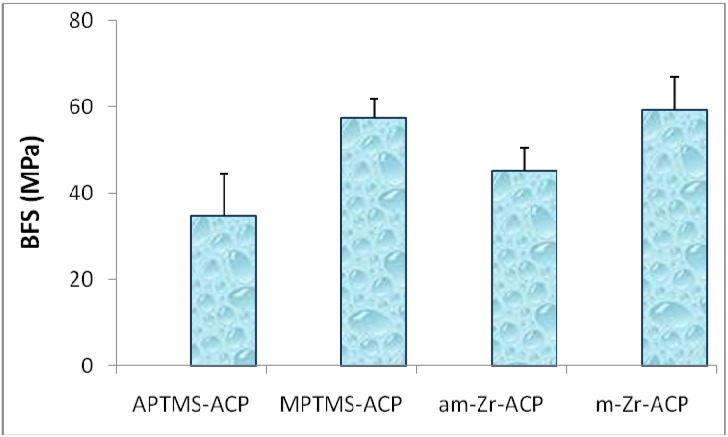
Effect of silanization and milling on the mechanical strength of ACP/ETHM composite specimens after 1 mo immersion in saline solution at 23 °C. Indicated are mean values + SD. Number of specimens n ≥ 5.

Additionally, ball milling of Zr-ACP prior to its utilization in composites was appraised as an alternative way to reduce the average size of the filler by breaking up large agglomerates into smaller particles that will more intimately interact with the resin and disperse more evenly in the composite. The composites were formulated with the same levels (40 mass%) of as-made (un-milled; am-Zr-ACP) and milled (m-Zr-ACP) and assessed for the mechanical strength in identical manner as silanized ACP composites. The BFS values of wet ETHM composite specimens prepared with MPTMS- and APTMS-silanized ACPs, and am- and m-Zr-ACP are presented in Figure 1. MPTMS- and m-Zr-ACP/ETHM composites attained significantly higher BFS than their APTMS- and am-Zr-ACP-based counterparts [(59.3 ± 7.6) MPa and (57.5 ± 4.4) MPa vs. [(34.8 ± 9.6) MPa and (45.3 ± 5.4) MPa, respectively. These results suggest that silanization of the ACP filler with MPTMS and milling has equal potential to ameliorate the overall plasticization of composites. While having no apparent effect on the structure, composition and/or morphology of the fillers, milling significantly reduced the average size of Zr-ACP particulates (from 5.9 μm to 0.9 μm) and the particle size range. Better dispersion of milled Zr-ACP in the resins resulted in the improved BFS of the composites after aqueous immersion. The physicochemical properties of am- and m-ACP composites are compared in Table 2.

The improvement in the BFS of m-ACP composites was accompanied with only a minimal reduction in their remineralizing ability (expressed as the thermodynamic stability of the immersion solutions containing the maximum concentrations of Ca and PO_4_ released from the disks with respect to stoichiometric HAP using the Gibbs free energy expression: ΔG^0^ = -2.303(RT/n)ln(IAP/K_sp_) [7,37]). The conclusion from the silanization and milling experiments is that it may be prudent to consider using MPTMS silanized or m-Zr-ACP when formulating remineralizing ACP composites for a wider spectrum of dental applications. 

It was recently reported [38] that silanization of the crystalline dicalcium phosphate anhydrous (DCPA) moderately increased strength while diminishingthe ion release from DCPA-filled Bis-GMA/TEGDMA composites. The authors assume that hydrophobic MPTMS coating on DCPA particles retarded the access of water to the particles thus slowing their dissolution. We believe that the more hydrophilic nature of the EBPADMA/TEGDMA/HEMA/MEP resin and the partially reduced affinity for water sorption of MPTMS-ACP particles compared to unsilanized ACP resulted in only minimally reduced Ca and PO_4_ release as observed in our ACP formulations.

**Table 2:** Results of the particle size analysis of the am-Zr-ACP and m-Zr-ACP fillers and the remineralizing capacity of their ETHM composites. *SD of mean values is indicated in parentheses.

Parameter	am-Zr-ACP	m-Zr-ACP
Particle size range (μm)	0.3 to 80.0	0.2 to 3.0
d_m_ (μm)	5.9 (0.7)*	0.9 (0.2)
Specific surface area, SSA (m^2^/g)	0.5 (0.1)	3.8 (1.0)
Ion activity product, IAP	99.26 (0.68)	101.21 (1.02)
Gibbs free energy, ΔG^0^ (kJ/mol)	-5.66 (0.21)	-5.07 (0.31)

**Figure 2:**
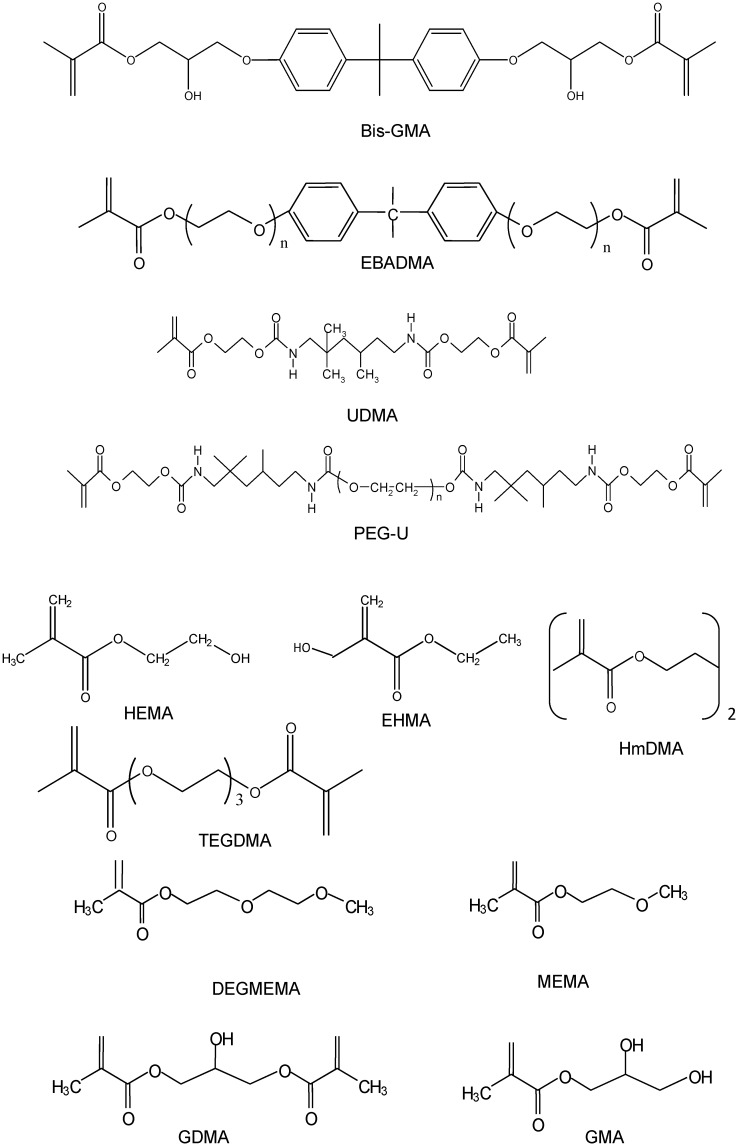

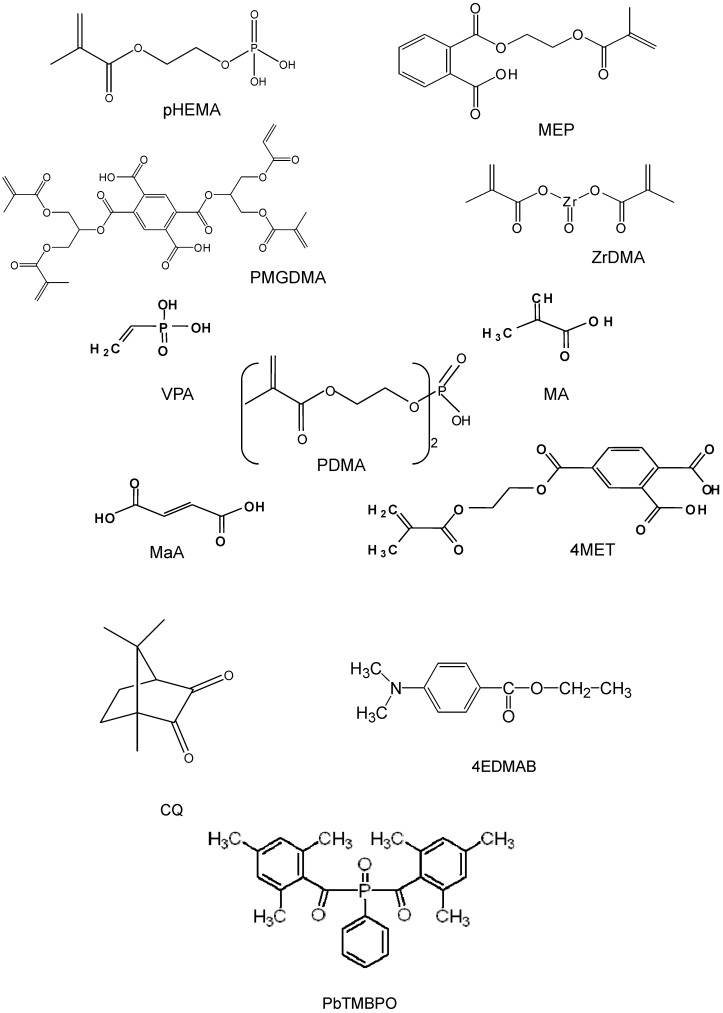
Chemical structure of the monomers and the components of the photo-initiating systems.

Methacrylate networks with good solvent resistance are widely used in dentistry as pit and fissure sealants, crown and bridge prostheses, dentinal bonding agents and tooth restorative composites [39]. Typical dental restorative resins are composed of a mixture of at least two dimethacrylate monomers: a relatively viscous base monomer and a low viscosity diluent comonomer. Monomers and components of the polymerization initiating systems discussed throughout this manuscript are listed in Table 3. Their chemical structures are presented in Figure 2 a-d.

The base monomer in the resin serves to minimize the PS by virtue of its relatively large molecular volume and enhance the modulus of the cured polymer, while the diluent monomer provides good handling properties and improves copolymer conversion due to its greater flexibility and smaller molecular volume [40]. 

The most commonly utilized copolymers are based on the base monomer Bis-GMA and the diluent monomer TEGDMA. The hydroxyl groups of Bis-GMA and the ethylene oxide segments of TEGDMA contribute to the relatively high WS of Bis-GMA/TEGDMA copolymers [41]. High concentrations of the more rigid structure of Bis-GMA typically result in monomer systems with relatively low DVC. PS, relatively low cure efficiency at ambient temperatures and plasticization of Bis-GMA/TEGDMA copolymers by oral fluids affect the service life of these composites. Alternative base monomers and/or diluent monomers have been explored to overcome some of the known shortcomings of the Bis-GMA/TEGDMA copolymers. 

Dental polymers based on EBPADMA, a relatively hydrophobic analog of Bis-GMA with a more flexible structure and lower viscosity, show higher degrees of cure and lower polymerization shrinkages than Bis-GMA/TEGDMA [42]. Urethane dimethacrylate (UDMA) usually reduces WS and PS while enhancing the mechanical properties of the resin matrices [7]. We hypothesize that complex resin matrices which comprise multifunctional monomers will improve matrix/ACP coherence and thus enable fine tuning the physicochemical properties of copolymers and their composites. The ultimate goal of the resin blending approach is to achieve a more optimal consistency suitable for the incorporation of particulate ACP fillers, increase the DVC upon curing and improve adhesion of the composite to tooth structures. The physicochemical basis for the expected interactions is a Lewis acid/base reaction in which the adhesive monomer is the electron donor and the ACP is the electron acceptor [43,44]. 

**Table 3:** Monomers and polymerization-initiating components used to formulate experimental resins discussed in the manuscript. Indicated acronyms are used throughout this manuscript. *Oligomer.

Component	Chemical Nomenclature	Acronym
Base monomers	2,2-bis[*p*-(2’-Hydroxy-3’-methacryloxypropoxy)phenyl]-propane	Bis-GMA
	Ethoxylated bisphenol A dimethacrylate	EBPADMA
	Urethane dimethacrylate	UDMA
Diluent monomers or oligomers	Di(ethyleneglycol)methyl ether methacrylate	DEGMEMA
	Ethyl-α-hydroxymethacrylate	EHMA
	Glyceryl dimethacrylate	GDMA
	Glyceryl monomethacrylate	GMA
	2-Hydroxyethyl methacrylate	HEMA
	Hexamethylene dimethacrylate	HmDMA
	2-Methoxyethyl methacrylate	MEMA
	Poly(ethylene glycol)-extended UDMA*	PEG-U
	Triethyleneglycol dimethacrylate	TEGDMA
Adhesive, surface-active monomers	Maleic acid	MaA
	Methacrylic acid	MA
	Methacryloyloxyethyl phthalate	MEP
	4-(Methacryloyloxy) ethyltrimellitate	4MET
	Bis[2-(Methacryloyloxy)ethyl] phosphate	PDMA
	Ethyleneglycol methacrylate phosphate	pHEMA
	Pyromellitic glycerol dimethacrylate	PMGDMA
	Vinyl phosphonic acid	VPA
	Zirconyl dimethacrylate	ZrDMA
Photoinitiating system	Camphorquinone	CQ
	Ethyl-4-*N,N*-dimethylaminobenzoate	4EDMAB
	Phenylbis(2,4,6-trimethylbenzoyl)phosphine oxide	PbTMBPO

A series of binary and ternary photo-activated resins using Bis-GMA, EBPADMA or UDMA as base monomer, and TEGDMA, HEMA or HmDMA as diluent monomers with distinctively different degrees of hydrophilicity were prepared and a battery of physicochemical tests were performed with copolymer and am-Zr-ACP composite specimens (Table 4). Bis-GMA- and EBPADMA-based copolymers, and to a lesser extent UDMA-based copolymers, attained higher DVC values when hydrophilic HEMA was included as a co-monomer in the resin, suggesting lower potential for the leaching out of unreacted monomeric species in these formulations. Higher DVCs for systems with relatively high HEMA content are attributed to HEMA’s monofunctionality and its high diffusivity. 

**Table 4:** Physicochemical evaluation of copolymers and ACP composites (bold) of binary and ternary Bis-GMA-, EBPADMA- and UDMA-based matrices containing HEMA or HmDMA with or without TEGDMA. Indicated values represent mean values; SDs are given in parenthesis. Number of samples in each experimental group: n ≥ 7 (DVC), n = 9 (PS), n ≥ 5 (WS), n = 3 (ΔG^o^) and n ≥ 8 (BFS). *Resin acronyms are derived from the first letters of the acronyms of the individual monomers, *i.e.,* Bis-GMA+ HEMA+HmDMA = BHHm.

Resin Matrix*	**DVC** (%)		**PS** (vol%)	**WS** (mass%)		**ΔG^o^** (kJ/mol)	**BFS** (mPa)	
							dry	wet
BH	78 (3)	**74 (2)**	**8.6 (2.6)**	3.2 (0.1)	**2.9 (0.3)**	**-4.20 (0.06)**	**63 (15)**	**61 (9)**
BHm	77 (2)	**67 (3)**	**5.4 (0.6)**	1.3 (0.2)	**2.2 (0.3)**	**-4.28 (0.20)**	**65 (13)**	**55 (10)**
BHT	77 (5)	**68 (4)**	**7.2 (0.9)**	4.8 (0.6)	**4.3 (0.6)**	**-4.43 (0.11)**	**65 (14)**	**40 (9)**
BHHm	81 (4)	**75 (2)**	**4.2 (1.0)**	3.3 (0.2)	**3.1 (0.2)**	**-4.70 (0.18)**	**71 (8)**	**48 (7)**
EH	82 (2)	**72 (2)**	**6.4 (0.7)**	1.3 (0.4)	**2.6 (0.2)**	**-5.86 (0.12)**	**61 (6)**	**53 (11)**
EHm	79 (2)	**69 (4)**	**7.2 (1.5)**	0.9 (0.2)	**2.6 (0.3)**	**-5.24 (0.11)**	**62 (8)**	**58 (7)**
EHT	85 (2)	**82 (1)**	**7.8 (1.5)**	3.8 (0.2)	**4.3 (0.5)**	**-5.75 (0.09)**	**59 (10)**	**49 (8)**
EHHm	84 (2)	**75 (1)**	**8.1 (1.1)**	2.0 (0.4)	**3.6 (0.2)**	**-6.58 (0.08)**	**58 (9)**	**50 (9)**
UH	77 (2)	**76 (1)**	**6.9 (0.8)**	2.2 (0.1)	**3.0 (0.4)**	**-4.24 (0.12)**	**61 (10)**	**57 (10)**
UHm	74 (2)	**74 (1)**	**6.6 (0.5)**	1.2 (0.3)	**2.6 (0.2)**	**-1.86 (0.13)**	**65 (8)**	**60 (12)**
UHT	84 (2)	**82 (1)**	**7.8 (0.8)**	3.5 (0.4)	**4.0 (0.6)**	**-4.36 (0.13)**	**54 (10)**	**40 (10)**
UHHm	83 (2)	**79 (3)**	**7.5 (0.8)**	3.3 (0.5)	**3.1 (0.5)**	**-4.48 (0.12)**	**63 (7)**	**37 (11)**

The PS measured in these experimental composites (4.2 vol% to 8.6 vol%) showed no apparent correlation with the resin formulation, and were higher than the PS values reported for the majority of commercial materials, most probably due to the lower filler content (mass fraction of only 40% ACP compared to an average mass fraction of 70% or more inert glass fillers in commercial composites). These high PS values are not surprising considering high DVC attained in the experimental composites (between 69% and 82%). It is also possible that the uneven dispersion of highly agglomerated am-Zr-ACP particles within the composite could have contributed to these high PS values. The range of PS values in experimental ACP composites falls into the category of either flowable composites or adhesive resins [(3.6 to 6.0)% and (6.7 to13.5)%, respectively [45]]. The excessive PS usually leads to stress development (PSS) both within the composite and at the tooth/restoration interface. An evaluation of PSS in composites is very important since the PSS may lead to marginal leakage and bacterial ingression leading to pulpal irritation, post restorative sensitivity and recurrent caries, which ultimately decrease the longevity of restoration. A number of material aspects (filler type and content, resin type and composition, type and concentration of initiators) and processing factors (ratio of the bonded to the unbounded (free) surface area of the composite in the cavity, *i.e.,* the configuration of C-factor) determine the PS, elastic modulus and PSS of the composite materials [45]. Although the PSS in polymeric methacrylate systems has been studied extensively [45,46,47,48,49,50,51,52], a fuller understanding of the relationship between the PS kinetics and the accompanying stress is still lacking. Recently, we have demonstrated that C-factor plays a key role in influencing the PSS of Bis-GMA/TEGDMA ACP composites [53]. We have also shown that inclusion of PEG-U oligomer into UDMA-based resins improved DVC while having no detrimental effect on PS, PSS and mechanical stability of composites [54]. Better performance of PEG-U-containing polymers compared to HEMA counterparts is attributed to the higher molecular mass and the more flexible character of PEG-U oligomer (due to a significant number of ethylene oxide units in its molecular structure) compared to less flexible poly(HEMA) segments in the matrix. 

**Figure 3:**
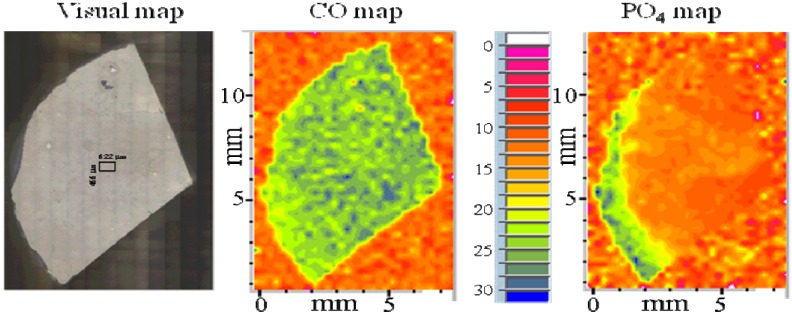
Distribution of the resin (CO map) and ACP (PO_4_ map) on a typical fragment of am-Zr-ACP/Bis-GMA/TEGDMA/HEMA composite disk specimen after aqueous immersion. The colors represent diminishing areas of CO and PO_4_ peaks: blue > green > yellow > orange > red.

In the case of ACP/methacrylate composites, not only water/polymer but also water/ACP interactions occur and both contribute significantly to the overall WS profiles. Besides affecting the strength (BFS usually decreases upon soaking), water sorption/diffusion influences the mineral ion release kinetics and, consequently, the remineralizing ability of these bioactive materials. The WS values of the binary and ternary Bis-GMA-, EBPADMA and UDMA copolymers and composites (consistently higher in HEMA- vs. the corresponding HmDMA-containing formulations; Table 4) were controlled by the content of hydrophilic (TEGDMA and HEMA) or hydrophobic (HmDMA) monomers in the resin matrix. There should be no doubt that water plays a very significant role in ACP filler-polymer matrix interactions. Whether it simply accelerates leaching out of the calcium and phosphate ions and thus induces filler failure or enhances polymer matrix plasticization, the excessive WS ultimately diminishes the mechanical stability of the composite material. Generally, dry ACP-filled composites had substantially lower BFS than unfilled specimens regardless of the type of filler or resin matrix. The strength of all ACP composites deteriorated upon exposure to the aqueous environment. Micro-FTIR mapping [9] of the water-immersed specimens revealed the existence of numerous defects/voids (resin-rich, phosphate-depleted regions) on their surfaces (Figure 3). 

The uneven distribution of ACP particulates throughout the matrix is, most probably, responsible for the inadequate filler/resin interlocking and the resulting adverse effect on the overall mechanical strength of composites. 

All binary and ternary Bis-GMA-, EBPADMA and UDMA-based composites were capable of releasing mineral ions at levels significantly above the minimum necessary for the re-precipitation of tooth mineral to occur (Table 4). The extent of the release was, however, affected by both the chemical structure and the composition of the monomer system (Table 4) as well as by the type of ACP filler (am- vs. m-Zr-ACP; Table 2). Generally, the anti-demineralizing/remineralizing capacity of ACP composites could be enhanced by: a) introducing EBPADMA as a base monomer, b) elevating the level of HEMA in the resin formulation and c) by utilizing am-ACP rather than m-ACP. The most probable mechanism by which the hydrophilic HEMA-enriched resins increased internal mineral saturation was by allowing the uptake of more water and/or better accessibility of ACP filler to the water already entrained. On the other hand, higher releases obtained with EBPADMA-based composites were attributed to a less cross-linked network of their resin matrix that produced a more open polymer matrices. When considering whether to use am-ACP or m-ACP in composite formulation, one has to consider the delicate balance between and the importance of the mechanical performance (which would require using m-ACP) vs. the expected remineralizing potential (which would favor am-ACP) for the intended application. 

**Figure 4:**
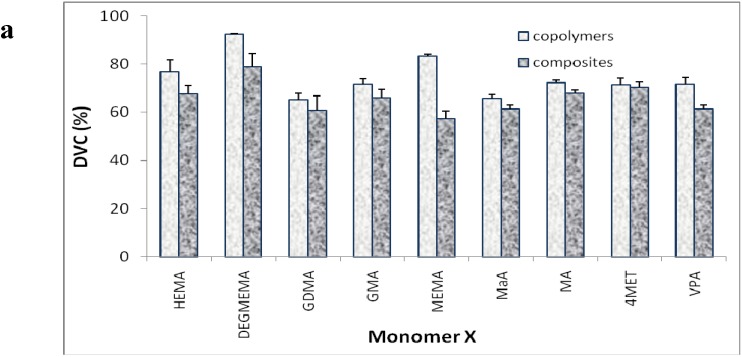

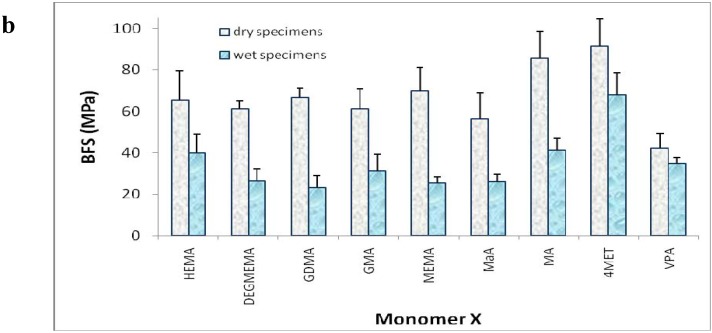
Degree of vinyl conversion (DVC) attained 24 h post-cure in ternary Bis-GMA/TEGDMA/X copolymers and their am-Zr-ACP composites (part a) and the biaxial flexure strength (BFS) of dry and wet (after two weeks in saline solution) composite specimens **(**part b**)**. Indicated are mean values with error bars indicating standard devation. Number of samples in each experimental group: n ≥ 3 (DVC), n = 5 (BFS).

To evaluate the effects of chemical and structural variations of the most hydrophilic monomer component (HEMA) and assess the effect of surface-active acidic co-monomers on DVC and mechanical properties of photo-activated BisGMA/TEGDMA/X copolymers and their composites, two series of experimental resins based on mixtures of Bis-GMA/TEGDMA (1:1 mass ratio) and HEMA-equivalent content of X on molar basis. First series comprised DEGMEMA, GDMA, GMA and MEMA, and the second series comprised MaA, MA, 4MET and VPA. The results are compiled in Figures 4 a, b. 

In the first series, DVC values attained with both copolymers and composites depended on resin composition and decreased in the following order: DEGMEMA > (HEMA, MEMA) > GMA > GDMA. In the second series, DVC of copolymers was practically unaffected by structural variations of the carboxylate comonomer for all but MaA formulations (mean DVC value for MA, 4MET and VPA = (72.3 ± 2.1)% vs. (65.3 ± 2.1)% for MaA). The monomer system based on DEGMEMA showed the highest DVC both in the copolymer and composite specimens. The addition of ACP generally resulted in a reduction of DVC. The differential in conversion for polymer and composite for the GMA- and GDMA-resins was significantly smaller than for the other resin systems. The differential in conversion for BT/acidic polymers and composites was generally smaller than for the ternary BT resins containing comonomers with hydroxyl or ether functionalities. No correlation could be established between DVC and the BFS values of the composites, dry or wet. In both series, DVC of composites was reduced compared to the corresponding copolymers. This reduction was lower with acidic, carboxylate-containing co-monomers (from 0.6% to 14.5%) than for a series of non-acidic, hydrophilic monomers with Bis-GMA/TGDMA resin (from 6.3% to 31.2%). The observed effect is most likely due to the decline in the exotherm of resin polymerization by the ACP filler phase, although other factors such as greater air entrapment and light scattering by ACP cannot be discarded as possible contributing factors to this reduction. The BFS of all dry composite specimens were on average (64.8 ± 8.7) MPa and (68.2 ± 12.0) MPa compared to (29.2 ± 6.3) MPa and (42.1 ± 6.3) MPa of all the wet composites in non-acidic, hydrophilic and acidic co-monomer series, respectively. The reduction in the mechanical strength of both types of composites after aqueous exposure is most probably caused by a reduction in intactness of ACP filler and rigidity of ACP/matrix interface caused by chemical and/or spatial changes that occurred during dissolution of ACP and its conversion to apatite. BFS measurements would suggest a need to reduce the level of hydrophilic monomer in order to improve the mechanical performance of the composites during aqueous exposure. To determine if there is a correlation between the observed reduction in mechanical strength and polymer matrix plasticization by water, extensive water sorption /desorption studies would be necessary. 

We have used hydrophilic HEMA, a widely used monomer in dental adhesive systems and in resin-modified glass ionomers, in the majority of our experimental formulations and showed that relatively high levels of HEMA in the matrix assure satisfactory ion release from ACP composites required for their remineralizing action. However, the inclusion of HEMA into experimental resins regularly resulted in elevated WS of copolymers and composites, and yielded composites that are mechanically weak. In an attempt to reduce the unwanted effects of HEMA on WS of composites, we assessed the effect of substituting EHMA (a unique isomer of HEMA [55]) for HEMA in Bis-GMA/TEGDMA/X (X = HEMA or EHMA) resins. Compared to HEMA, EHMA is more hydrophobic and significantly less soluble in water, probably as a result of the different patterns of hydrogen bonding in two isomers. Hypothetically, EHMA-containing resins are expected to show considerably more intra-molecular than inter-molecular hydrogen bonding thus having lower water solubility. The copolymers formulated with more hydrophilic HEMA adsorbed on average 37% more water compared with less hydrophilic EHMA. However, in ACP composites these differences were only marginal due to the contribution of highly hygroscopic ACP filler to the overall water sorption. Significantly, attained levels of DVC in both formulations were practically identical (85% in copolymers and 81% in composites). In aqueous environment, both formulations yielded solutions highly supersaturated with respect to hydroxyapatite indicating that substituting EHMA for HEMA in ternary Bis-GMA/TEGDMA/X matrices did not compromise the remineralizing potential of composites. In light of these results, EHMA can be considered as a suitable substitute for HEMA in dental applications. 

The adhesiveness to tooth structures of the experimental ACP composites is expected to be achieved by the inclusion of surface-active, hydroxyl-containing methacrylate (primarily HEMA, its isomer EHMA, GDMA or GMA), carboxylate- (MaA, MA, MEP, 4MET, PMGDMA), phosphate- (PDMA, pHEMA) or phosphonate-(VPA) containing monomers (Table 3). Phosphate-containing monomers in equivalent molar mixtures Bis-GMA:X or Bis-GMA:TEGDMA:X = 1:1.8 and 1:1.8:3.1 for binary and ternary systems, respectively (X = PDMA or pHEMA), when formulated with am-Zr-ACP into composites exhibited spontaneous gelation which was followed by solidification before the samples were light-cured. FTIR analysis (changes in ν_1_ and ν_3_ as well as ν_4_ phosphate domains) suggested that the observed phenomenon was due to the specific interactions between the ACP filler and PDMA or pHEMA. At these relatively high concentrations, both pHEMA and PDMA also had a deleterious effect on both the water sorption (WS) and the BFS of the composites [unpublished data]. These monomers could only be used in future formulations at significantly reduced concentrations to avoid premature self-hardening of the experimental composites. Both non-acidic, hydrophilic (DEGMEMA, GMA, GDMA, MEMA) and acidic co-monomers (MaA, MA, 4MET, VPA) yielded copolymers and/or composites that were not superior to the HEMA counterparts and were not yet further assessed. Inclusion into the resin of PMGDMA and/or MEP [56] could compromise the remineralizing ability of the composites due to the excessive Ca-ion binding seen in these systems. The affinity of carboxylate functionalities of the surface active monomer to bind free Ca ions in the solution would suggest keeping the concentration of such monomer in the resin at the minimum level needed to achieve the desired adhesion effect.

**Figure 5:**
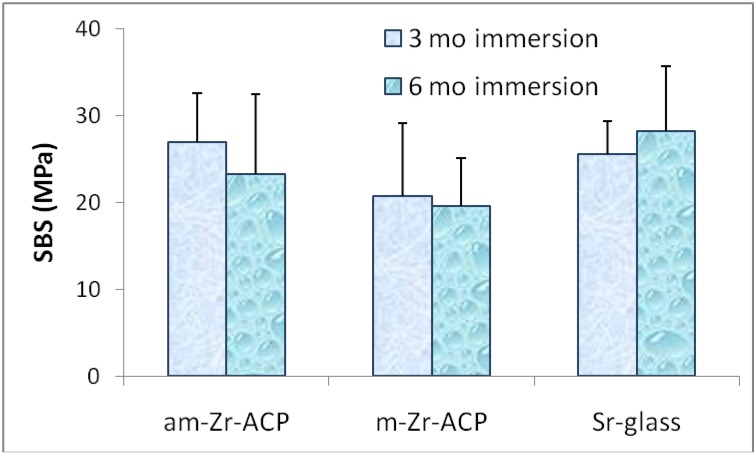
Effect of filler type on shear bond strength (SBS) of Bis-GMA/TEGDMA/HEMA/ZrDMA composites immersed in distilled water for 3 mo and 6 mo at 37 °C. Indicated are mean values + standard deviation of the minimum of seven specimens in each experimental group.

Shear bond strength (SBS) testing of am-Zr-ACP composites formulated with BisGMA/TEGDMA/HEMA/ZrDMA (BTHZ) and PMGDMA/TEGDMA (PT) resins [57] revealed that the strength of composite/adhesive/dentin bond was practically unaffected by the composition of the two experimental resins [the average SBS value was (18.3 ± 3.5) MPa]. SBS values of specimens prepared with am- and m-Zr-ACP were also unaffected by either the filler type or the immersion type for up to six months (Figure 5). It would appear that am- and m-ACP fillers, despite the flaws they introduce into the microstructure of the composites, do not adversely affect the short- and mid-term bonding behavior of the composite to an adhesive resin. Rather, they perform at least as well as glass-reinforced composites, while providing an additional bioactive component that may promote remineralization in dentin, enamel or both. Future SBS studies that will be carried out with the EBPADMA- and UDMA based matrices formulated for orthodontic and endodontic application, respectively, are expected to provide further evidence on whether it is possible to formulate ACP composites with improved bonding performance without compromising their biocompatibility, remineralizing potential or both. 

## 3. Experimental Section 

### 3.1. ACP filler synthesis and characterization

ACP was synthesized as originally reported in ref [15]. Experimental protocols pertinent to surface-modification of ACP are detailed in our earlier publications [7,31,32]. Silanization of ACP was performed as follows: 3-aminopropyltrimethoxysilane (APTMS) and methacryloxypropyltrimethoxy- silane (MPTMS) were applied at 2 mass% relative to Zr-ACP. To catalyze the hydrolysis of the methoxy groups, MPTMS was deposited from an aqueous/alcohol (5/95 vol%) solution adjusted to pH 5.5 by addition of acetic acid. For APTMS acidification was not necessary, since its amino group auto-catalyzed the hydrolysis/condensation reaction. Five minutes was allowed for completion of hydrolysis and silanol formation following initial mixing-in of the ACP powder and stirring for 30 min. The pH of the MPTMS/ACP slurry was then adjusted to 10 by the addition of 100 mmol/L KOH solution to facilitate the condensation and formation of siloxanols. After filtration and drying at room temperature, the silanized ACP was heated at 100 °C for 30 min to strengthen the coating by secondary formation of polylsiloxane network structures. Unbonded silane molecules were washed-out with ethanol and the silane-coated ACPs, same as all the other types of ACPs, were kept dry in a desiccator until used for composite preparation. The amorphous state of ACPs was verified by powder X-ray diffraction (XRD; Rigaku DMAX 2000 X-ray diffractometer; Rigaku/USA Inc., Danvers, MA, USA) and Fourier-transform spectroscopy (FTIR; Nicolet Magna-IR FTIR 550 spectrophotometer; Nicolet Instrumentations, Madison, WI, USA). XRD patterns were recorded from 4° to 60° 2θ with CuKα radiation (λ = 0.154 nm) at 40 kV and 40 mA. The samples were step-scanned in intervals of 0.010° 2θ at a scanning speed of 1.000 deg/min. The FTIR spectra (4,000 cm^-1^ to 400 cm^-1^) were recorded using a KBr pellet technique (0.8 mg to1.0 mg solid/400 mg KBr). The FTIR micro-spectroscopy (FTIR-m; a Nicolet Magna-IR 550 FTIR spectrophotometer equipped with a video camera, a liquid nitrogen cooled-mercury cadmium telluride detector, a computerized motorized mapping stage and the Omnic Atlus software) was utilized to produce functional group maps in conjunction with video images of intact copolymer and composite surfaces as well as cross-sections of copolymer and composite specimens before and after exposure to aqueous environments [9]. D.1.3. Particle size distribution (PSD) of the fillers was measured using a centrifugal particle size analyzer (SA-CP3; Shimadzu Scientific Instruments Inc., Columbia, MD, USA). ACP powders were dispersed in isopropanol and utrasonicated for 10 min at room temperature prior to the analysis (a minimum of three runs for each experimental group). From the PSD(s), the median particle size diameter (d_m_) and the corresponding specific surface area (SSA) of the sample will be obtained. Changes in d_m_ are taken as a primary indicator of the state of agglomeration of the ACP particulates (the higher the d_m_ value, the more aggregated the ACP). The PSD data were routinely compared with morphology/topology evaluations (scanning electron microscopy (SEM): JSM-5400 instrument, JEOL Inc. Peabody, MA, USA). The overall water content (mass fraction, %) and the ratio of surface-bound vs. structurally incorporated water of ACP fillers was determined by thermogravimetric analysis (TGA; Series 7 Thermal Analysis System, Perkin Elmer, Waltham, MA, USA). 

### 3.2. Resin formulation, copolymer and composite specimen preparation

The experimental resins were formulated from commercially available monomers which were used as received from the manufacturer, *i.e.,* without additional purification. All but Bis-GMA/TEGDMA/4MET resin were photo-activated with CQ (0.2 mass%) and 4EDMAB (0.8 mass%). In 4MET-containing resin, PbTMBPO (1.96 mass%) was utilized as the photo-initiator because of storage stability problems encountered with the use of CQ and 4EDMAB with the 4MET monomer. After introducing the appropriate initiators to the monomer blends, the activated resin mixtures were magnetically stirred until fully homogenized 

The composite pastes were prepared by hand spatulation by combining mass fractions of 40% ACP filler and 60% resin. The volume filler fraction of the experimental material was approximately 30%. Its shade is B1 compared to the VITAPAN Lumin Vacuum shade guide (Vita Zahnfabrik, Bad Säckingen, Germany). The observed slightly higher translucency is attributed to the relatively low filler content by volume compared to commercial composites. Control composite specimens used in bond strength measurements were made with the unsilanized Sr glass (Dentsply Caulk, Milford, DE, USA). To achieve a consistency comparable to ACP composites, a mass fraction of 70% Sr-glass was blended with the resin in these control specimens. Copolymer and composite specimens alike were cured by irradiating disk-shaped specimens (cylindrical molds with an average 13 mm diameter and 1.5 mm thickness) for 60 s each side (Triad 2000; Dentsply International, York, PA, USA).

### 3.3. Polymerization shrinkage (PS)

The PS of composite specimens was measured by a computer-controlled mercury dilatometer developed at the PRC-ADAF, Gaithersburg, MD, USA [8]. Composite pastes were cured using a standard 60 s + 30 s exposure and data acquisition of 60 min + 30 min. A minimum of triplicate runs were performed for each experimental group. PS of each specimen corrected for temperature fluctuations was plotted as a function of time. The overall PS (volume fraction, %) was calculated based on the known mass of the sample (50 to 100 mg) and its density. The latter was determined by means of the Archimedean displacement principle using an attachment to a microbalance (YDK01 Density Determination Kit; Sartorius AG, Gőttingen, Germany). 

### 3.4. Polymerization stress (PSS) measurements

PSS was quantified by utilizing a computer-interfaced, cantilever beam tensometer developed at the Paffenbarger Research Center, American Dental Association Foundation, Gaithersburg, MD, USA [53,54]. The deflection of the cantilever beam was measured with a linear variable differential transformer. The force was calculated from a beam length (12.5 cm) and a calibration constant (3.9 N/μm). PSS was obtained by dividing the measured force by the cross sectional area of the sample (diameter = 6 mm). The composites were irradiated through the lower quartz rod with a visible light (Spectrum Curing Unit, Dentsply-Caulk, Milford, DE, PA, USA) for 60 s to initiate polymerization, and the PSS was then measured after 60 min. The light intensity, measured by a Demetron Model 100 radiometer (Demetron Research, Danbury, CT, USA) was (510 ± 25) mW/cm^2^ at the upper end of the top quartz rod where the sample was bonded.

### 3.5. Degree of vinyl conversion (DVC)

The DVC of copolymer and composite specimens was measured at 23 °C by near-infrared (NIR) spectroscopy [58]. NIR scans (Nicolet Magna 550, Nicolet Inc., Madison, WI, USA) were taken before photo cure and 24 h post-cure of composites with a thickness of approx. 3.0 mm and compared. DVC was defined as the% change in the integrated peak area of the 6165 cm^-1^ absorption band related to the first overtones of the = C-H stretching vibrations of the methacrylate vinyl group (= CH_2_) before and after photo-polymerization. It was calculated utilizing the following formula:

DVC = {1-[(area/thickness)_polymer_/(area/thickness)_monomer_]} × 100 (1)

By measuring the thickness of monomer/polymer specimens, the need to use an invariant absorption band as an internal standard was circumvented. 

### 3.6. Biaxial flexure strength (BFS)

The BFS values of copolymer and experimental composite disk specimens were determined by using a computer-controlled Universal Testing Machine (Instron 5500R, Instron Corp., Canton, MA, USA) operated by Testworks 4 software, and a piston-on-three-ball loading arrangement (suitable for even slightly warped specimens). The BFS values were calculated according to the following equation [59]:

BFS = AL/t^2^ (2)

where A = -[3/4π(X-Y)], X = (1+ν)ln(r_1_/r_s_)^2^ +[(1-ν)/2](r_1_/r_s_)^2^, Y = (1+ν)[1 + ln(r_sc_/r_s_)^2^], ν = Poisson’s ratio, r_l_ = radius of the piston applying the load at the surface of contact, r_sc_ = radius of the support circle, r_s_ = radius of disk specimen, L = applied load at failure, and t = thickness of disk specimen. 

### 3.7. Water sorption (WS)

The WS of copolymer and/or composite specimens was determined as described below. Specimens were initially dried over CaSO_4_ until a constant mass was achieved (± 0.1 mg) and then exposed to an air atmosphere of 75% relative humidity (RH) at 37 °C by keeping them suspended over saturated aqueous NaCl slurry in closed systems. Gravimetric mass changes of padded dry specimens will be recorded at predetermined time intervals. The degree of WS of any individual specimen at a given time interval (t), expressed as a% mass fraction, was be calculated using a simple equation:

WS = [(W_t_ – W_o_)/W_o_] × 100 (3)

where W_t_ represents the sample mass at the time t, and W_o_ is the initial mass of dry sample.

### 3.8. Ion release from composites

Mineral ion release from ACP composite disk specimens was assessed at 23 °C, in magnetically stirred, HEPES-buffered saline solutions (25 mL saline/specimen). Saline solution was replaced at predetermined time intervals. The kinetic changes in calcium and phosphate concentrations were determined by utilizing atomic emission spectroscopy (Prodigy High Dispersion ICP-OES, Teledyne Leeman Labs, Hudson, NH, USA). 

### 3.9. Shear bond strength (SBS)

Adhesive bonding to dentin was measured on extracted human molars and pre-molars embedded with cold-cured resin in poly-carbonate cups. Exposed dentin surfaces of the tooth samples were ground flat at a 90° angle to the longitudinal axis of the polycarbonate holder. Moist dentin surfaces were primed sequentially with *N*-phenylglycine/acetone and PMGDMA/acetone solutions and photo-activated. A polytetrafluoroethylene-coated ring (4 mm in diameter and 1.5 mm in thickness) was used as a mold for the composite. Teflon tape (0.3 mm thick) with a hole coinciding with the hole in the ring was placed under the ring to prevent the ring from adhering to the dentin. Both the ring and the tape were placed in the center of the dentin surface and held down with a lead weight (450 g). The cavity in the ring was filled with the composite, irradiated for 20 s with a commercial visible-light source. Specimens were completed by applying a resin-based composite (TPH, Dentsply Caulk, Milford, DE, USA) and then stored (at 37 °C) immersed in distilled water for up to 6 months prior to de-bonding. Samples were de-bonded by placing the assembly against the vertical surface of a nylon block and the ring-enclosed composite was sheared off at a cross-head speed of 0.5 mm/min with a flat chisel pressing against the edge of the brass ring and connected to the load cell of the testing machine. 

### 3.10. Statistical data analysis

Experimental data were analyzed by analysis of variance (ANOVA; α = 0.05). Significant differences between the groups were determined by all pair-wise multiple comparisons (Tukey-test). One standard deviation (SD) is identified in this paper for comparative purposes as the estimated uncertainty of the measurements. 

## 4. Conclusions 

Chemical structure and composition of the methacrylate monomers used to form the matrix phase of amorphous calcium phosphate (ACP) composites significantly affected degree of vinyl conversion (DVC) and water sorption (WS) of both copolymers and composites and the release of mineral ions from the composites. Modification of ACP surface via introducing cations and/or polymers *ab initio* during filler synthesis failed to yield mechanically improved composites. However, moderate improvement in composite’s mechanical stability without compromising its remineralization potential was achieved by silanization and/or milling of ACP filler. Combining ethoxylated bisphenol A dimethacrylate (EBPADMA) or urethane dimethacryalte (UDMA) as base monomers and adding moderate amounts of hydrophilic 2-hydroxyethyl methacrylate (HEMA) or its isomer ethyl-α-hydroxy- methacrylate (EHMA) appears to be a promising route to maximize remineralizing ability of the filler while maintaining high DVC (an indirect indicator of high biocompatibility). However, additional adjustments in resin formulations may be necessary to improve the polymerization shrinkage and stress that develops in experimental ACP composites as currently formulated. Inclusion into the resin of the surface active monomers with phosphate functionalities (bis[2-methacryloyloxy)ethyl] phosphate (PDMA) and ethyleneglycol methacrylate phosphate (pHEMA)) resulted in composites that did not meet the basic physicochemical requirements for useful ACP composites. When formulating resins that include adhesion-promoting monomers in the resins, the levels of these monomers should be kept low enough to avoid excessive binding of the released Ca ions by their carboxylate groups (depletion of Ca leads to diminished remineralizing potential). Resin fine-tuning and filler modification should be considered in designing composites intended for a variety of dental applications. A delicate balance between the DVC, PS and PSS, as well as between the WS, mechanical stability and ion release, needs to be established before these remineralizing materials could be recommended for clinical evaluation. 

## Appendix. List of Acronyms Used throughout the Manuscript

ACP: amorphous calcium phosphate
ADAF: American Dental Association Foundation
am-ACP: as made ACP
ANOVA: analysis of variance
APTMS: 3-aminopropyltrimethoxysilane
BFS: biaxial flexural strength
BH: Bis-GMA/HEMA resin
BHHm: Bis-GMA/HEMA/HmDMA resin
BHm: Bis-GMA/HmDMA resin
BHT: Bis-GMA/HEMA/TEGDMA resin
Bis-GMA: 2,2-bis[*p*-(2-hydroxy-3-methacryloxypropoxy)phenyl]propane
CQ: camphorquinone
DCPA: dicalcium phosphate anhydrous
DEGMEMA: di(ethyleneglycol)methyl ether methacrylate
d_m_: median diameter
DVC: degree of vinyl conversion
EBPADMA: ethoxylated bisphenol A dimethacrylate
4EDMAB: ethyl-4-*N,N*-dimethylamino benzoate
EH: EBPADMA/HEMA resin
EHHm: EBPADMA/HEMA/HmDMA resin
EHm: EBPADMA/HmDMA resin
EHMA: ethyl-α-hydroxymethacrylate
EHT: EBPADMA/HEMA/TEGDMA resin
ETHM: EBPADMA/TEGDMA/HEMA/MEP resin
FTIR: Fourier transform infrared spectroscopy
FTIR-m: FTIR microspectroscopy
ΔG^0^: Gibbs free energy
GDMA: glyceryl dimethacrylate
GMA: glyceryl monomethacrylate
HAP: hydroxyapatite
HEMA: 2-hydroxyethyl methacrylate
HEPES: 4-(2-hydroxyethyl)-1-piperazineethanesulfonic acid
HmDMA: hexamethylene dimethacrylate
IAP: ion activity product
MaA: maleic acid
MA: methacrylic acid
m-ACP: milled ACP
MEMA: 2-methoxyethyl methacrylate
MEP: methcryloyloxyethyl phthalate
4MET: 4-(methacryloyloxy) ethyltrimellitate
MPTMS: methacryloxypropyl trimethoxy silane
NIDCR: National Institute of Dental and Craniofacial Research
NIR: near infrared spectroscopy
NIST: National Institute of Standards and Technology
PAA: poly(acrylic acid)
PbTMBPO: phenylbis(2,4,6-trimethylbenzoyl)phosphine oxide
PDMA: bis[2-(methacryloyloxy)ethyl] phosphate
PEG-U: poly(ethylene glycol) extended urethane dimethacrylate
PEO: poly(ethylene oxide)
pHEMA: ethyleneglycol methacrylate phosphate
PMGDMA: pyromellitic glycerol dimethacrylate
PRC: Paffenbarger Research Center
PS: polymerization shrinkage
PSS: polymerization stress
PSD: particle size distribution
RH: relative humidity
SBS: shear bond strength
SEM: scanning electron microscopy
SD: standard deviation
SSA: specific surface area
TEGDMA: tri(ethyleneglycol) dimethacrylate
TGA: thermogravimetric analysis
UDMA: urethane dimethacrylate
UH: UDMA/HEMA resin
UHHm: UDMA/HEMA/HmDMA resin
UHm: UDMA/HmDMA resin
UHT: UDMA/HEMA/TEGDMA resin
UPHM: UDMA/PEG-U/HEMA/MEP resin
VPA: vinyl phosphonic acid
WS: water sorption
XRD: X-ray diffraction
ZrDMA: zirconyl dimethacrylate
